# Intra-Individual Variability in Gross Motor Development in Healthy Full-Term Infants Aged 0–13 Months and Associated Factors during Child Rearing

**DOI:** 10.3390/children9060801

**Published:** 2022-05-30

**Authors:** Rungreudee Tupsila, Wantana Siritaratiwat, Surussawadi Bennett, Lugkana Mato, Orawan Keeratisiroj

**Affiliations:** 1 Research Center in Back, Neck, Other Joint Pain and Human Performance (BNOJPH), Faculty of Associated Medical Sciences, Khon Kaen University, Khon Kaen 40002, Thailand; thupsila@gmail.com (R.T.); surmac@kku.ac.th (S.B.); 2 School of Physical Therapy, Faculty of Associated Medical Sciences, Khon Kaen University, Khon Kaen 40002, Thailand; yui@kku.ac.th; 3 Faculty of Public Health, Naresuan University, Phitsanulok 65000, Thailand; orawansa.nu@gmail.com

**Keywords:** gross motor development, Alberta Infant Motor Scale, full-term infants, intra-individual variability, infant walker, baby hammock

## Abstract

The gross motor development of a typically developing infant is a dynamic process, the intra-individual variability of which can be investigated through longitudinal assessments. Changes in gross motor development vary, according to the interaction of multiple sub-systems within the child, environment, task setting, and experience or practice of movement. At present, studies on environmental factors that influence gross motor development in full-term infants over time are limited. The main aim of this study was to investigate environmental factors affecting intra-individual variability from birth to 13 months. The gross motor development of 41 full-term infants was longitudinally assessed every month from the age of 15 days using the Alberta Infant Motor Scale. Parents were interviewed monthly about environmental factors during childcare. Infants showed fluctuations in the percentile of gross motor development, and no systematic pattern was detected. The total mean range of gross motor percentile was 65.95 (SD = 15.74; SEM = 2.28). The percentiles of gross motor skills over the 14 assessments ranged from 36 to 93 percentile points. Factors that were significantly associated with the gross motor development percentile were the use of a baby walker (Coef. = −8.83, *p* ≤ 0.0001) and a baby hammock (Coef. = 7.33, *p* = 0.04). The use of baby hammocks could increase the gross motor percentile by 7.33 points. Although the usage of a baby walker is common practice in childcare, it may cause a decrease in the gross motor percentile by 8.83 points according to this study. In conclusion, healthy full-term infants exhibited a natural variability in gross motor development. Placing infants in a baby walker during the first year of age should be approached with caution due to the risk of delayed gross motor development.

## 1. Introduction

The screening of gross motor development during the first two years of life is a common practice that allows for the timely detection of developmental delays or deviations. It is accepted that development during infancy and childhood is a dynamic process and, therefore, the onset of gross motor milestones can vary among typically developing full-term infants. The World Health Organization (WHO) has conducted a longitudinal multi-center study to establish windows of achievement of key gross motor developmental milestones among healthy full-term infants from five countries. The results of the study showed that the ages at which key motor milestones are achieved display normal variation among healthy infants [1]. About 90% of healthy infants achieved key gross motor milestones in a particular sequence, starting from crawling and progressing to sitting without support, standing with assistance, walking with assistance, standing alone, and, finally, walking alone, whereas 4.3% of the infants were non-crawlers [1].

According to the dynamic systems approach, the changes and appearance of gross motor skills within an individual infant are a result of an interaction of sub-systems within the child, the environment, and the setting of the task [2]. Developmental surveillance is, therefore, recommended in clinical practice [3]. Research on longitudinal standardized assessments has revealed variation in the emergence of gross motor skills among typically developing full-term, low-, and high-risk infants over the first year of life [4,5,6,7]. In a longitudinal study, Darrah et al. observed gross motor development in healthy full-term infants, and found that the motor development percentile within typically developing infants was not stable throughout the first 13 months [4]. Later, in 2003, Darrah et al. used the Peabody Developmental Motor Scales and the Communication Symbolic Behavior Scales Developmental Profile to perform a longitudinal assessment of the development of infants in their homes at 9, 11, 13, 16, and 21 months of age. Typically developing infants showed a non-linear (rather than constant) rate of fine motor, gross motor, and communication development [5]. Furthermore, Lobo et al. found that there was instability in the delay classification of a standard assessment, therefore suggesting that clinical judgment should consider parent reports and naturalistic assessment or observation of spontaneous movement, participation, and social interaction with peers across contexts [7].

The role of environmental factors and movement experiences in infant development is as important as (or possibly more important than) biological factors [8,9]. Movement experiences during development [10,11] and the environment can promote or hinder gross motor development. For instance, there has been controversy about how lying in a supine position could delay gross motor development. This could be because while awake, supine can limit the opportunities for infants to practice anti-gravity movements and postural control in extension [8,12,13,14], resulting in delayed gross motor development in upright posture [15,16]. Darrah et al. argued that 67.2% of infants in their study were placed to sleep in a supine position, while the remaining participants were placed in different postures; nevertheless, none of them showed delay in gross motor development [17]. Parents reported that healthy infants could roll in both supine and prone positions independently, and it is difficult to keep infants in one position while they are awake. Having opportunities or experiences in upright positions, such as being carried, supported in the mother’s arms, being seated during daily childcare, or placing infants in positions with movement independence promotes head and postural control [9,10,18]. Moreover, using various equipment or toys during daily activity can have either a positive [9] or negative [19,20,21,22,23,24,25,26] effect on gross motor development. Furthermore, equipment use was found to be inversely related to the motor development of infants [19].

Previous cross-sectional studies have found that factors associated with motor development include parental education [8,27,28,29,30], household income [8,27,29], parental employment [30], duration of maternal leave [30,31], family characteristics [6,29,30], space for physical activity [8], sleep position [15,20], and equipment usage [19,21,22,32]. A systematic review, however, concluded that longitudinal research on factors associated with gross motor development is required [33]. Although time-consuming and costly, a longitudinal follow-up could provide empirical evidence of the impact of the environment on motor development and reflect the dynamic variance in gross motor development. A literature review shows only a longitudinal study on variability in gross motor development in healthy infants and the effects of daily activities and home environmental factors [9]. Therefore, the objectives of this study were to: (1) investigate the intra-individual variability in gross motor development in infants from birth to 13 months and (2) examine the effect of environmental circumstances on the intra-individual variability in gross motor development.

## 2. Methods

### 2.1. Study Site and Participants

This study used a longitudinal design and was conducted at the homes of infants. The population consisted of newborn infants between June and December 2017. Name lists of newborns were requested from 26 district health-promoting hospitals within a range of 50 km from Khon Kaen University, Thailand. In order to obtain a representative sample, systematic random sampling was used. The researchers randomly selected every two infants from the first to the last name of newborns. Thereafter, the parents of selected infants were contacted, and an appointment was made for an explanation of the research and to obtain informed consent. Families could leave the research study whenever desired without any effect on their medical services.

As no longitudinal study regarding the effect of environment on Thai child-rearing culture exists, the sample size calculation in the study was carried out using the standard deviation of the AIMS scores from a previous study conducted in home-raised healthy full-term infants aged 8 months [34]. The widest standard deviation of 4.3 was used to calculate the number of participants, such that the required number of participants could cover the variation in gross motor development of the population. The appropriate sample size was determined as 41 infants. However, the sample size was re-checked after data collection was completed, by calculating the post hoc power of testing.

The inclusion criteria were healthy full-term infants born between 37 and 40 weeks, of Thai nationality, had a birth weight of more than 2500 g, had an Apgar score at 5 min of 9 or 10, and who were being raised by parents or close relatives at home. Infants were excluded if they were blind or deaf; were diagnosed with a congenital abnormality, had abnormal muscle tone or persistent jerky movement after the first few weeks of birth, had a history of perinatal complications such as premature birth and/or low birth weight, delivery using vacuum extraction, or had any acute or chronic illnesses within 7 days before assessment. To prevent the collection of redundant data, twins were excluded from the study. Infants whose mother had a history of mental problems, such as depression and suicidal ideation, or physical problems, such as asthma and hypertension, were also excluded. Infants who participated in fewer than 12 assessments of their gross motor development or who went to a nursery or daycare center were also excluded from the data analyses. A name list of 54 newborns was obtained from the systematic selection. Three newborns were excluded due to premature birth, and seven families declined to participate in the research. A total of 44 remaining infants were recruited in the study.

### 2.2. Instrument

The Alberta Infant Motor Scale (AIMS) is an observational tool, which was used in this study to examine the gross motor development of infants. The AIMS can be used to evaluate infants from birth to 18 months, or when they display independent walking, and focuses on four main positions distributed into 58 items: 21 in prone, 9 in supine, 12 in sitting, and 16 in standing [35]. Each item includes components of weight-bearing, posture, and anti-gravity movements. A score of 1 point is given for an “observed” item, and 0 points are assigned for a “not observed” item. The scores of the least and the most mature observed abilities in each main position represent the window of gross motor performance in each position. Each item below the least mature observed ability is given 1 point; these are considered the previous items credited. The observed items and the previous items credited are summed for each main position. The total score for all 4 positions is out of a maximum of 58 [35]. The assessment takes approximately 30 min for each infant. The cutoff reference points for delayed motor development used in the study are below the 10th percentile at an age of 4 months and below the 5th percentile at an age of 8 months [36].

### 2.3. Outcome Measures

To investigate the intra-individual variability of gross motor development, the gross motor development of each infant was longitudinally assessed every month, using the Thai version of the AIMS [37], from the age of 15 days until 13 months, or until the onset of independent walking. In this study, independent walking was defined as the ability to voluntarily walk at least five steps without support [38].

To examine the factors related to the variability of gross motor percentiles, parents or caregivers were interviewed about socio-economic factors, sleep position, and the usage of childcare equipment. Demographic data, including gender, gestational age, weight and height at birth, head circumference, Apgar score at 5 min, delivery methods, and birth order, were recorded when the first data collection cycle was performed. Each type of equipment usage was recorded as used (yes) or non-used (no), and results are reported in percentages.

### 2.4. Procedure

Ethics approval to conduct the study was obtained from the Khon Kaen University Ethics Committee for Human Research based on the Declaration of Helsinki and the ICH Good Clinical Practice Guideline (Institutional Review Board Number: IRB00008614, Protocol ID No: HE612144, 17 May 2018). Parents provided their informed consent to voluntarily participate and allowed their infants to be observed for gross motor development before the first data collection cycle. Throughout the study period, participants could withdraw from the study at any time, if desired. The data were anonymized, in order to not reveal the identities of participants, and the analyses were conducted in such a way that prevented the final results from being linked to individuals.

The eligibility of participants for the study was assessed by an interview, data recording, and direct observation by a licensed pediatric physical therapist. Demographic data of infants were obtained from child health record books and recorded prior to the first data collection. Parents or caregivers were also interviewed every month, using structured questionnaires about childcare during the data collection period. Moreover, logbooks were provided for the family to record information about illnesses experienced by the infant, such as signs, duration of illness, and treatment. Vaccination data were obtained from the child health record book, in order to ensure that infants had received the required healthcare.

The gross motor development of each infant was longitudinally assessed every month for 14 times in total. Subsequent assessments occurred on the same date of every month, plus or minus five days. The assessments of motor development were performed through direct observation at home, in a quiet room familiar to the infant, and with the parents or main caregiver nearby. Each infant wore a diaper during the assessment, such that his or her joints and movements could be clearly observed. Infants moved voluntarily with minimal touching. The parents or main caregivers were asked to change the position of the infants, if needed. Toys could be used to motivate movement, if necessary. The assessment was performed only when infants or toddlers were alert or felt well. The assessment of gross motor development was re-scheduled for another time within five days of the due date if infants were not ready for the test.

Only one physiotherapist assessed participants in the current longitudinal study. The assessor was trained to use the AIMS Thai version, and performance was determined by an expert with more than 10 years of clinical experience in pediatric physical therapy and who is familiar with the AIMS Thai version. The inter-rater reliability between the assessor and expert was tested and analyzed using the intra-class correlation coefficient (ICC (3,1)). The result was 0.991 (95% CI 0.983–0.996). The intra-rater reliability with a 1-month interval was assessed in 25 full-term infants aged 0–18 months who were not included in the current study; the ICC (3,1) value was 0.979 (95% CI = 0.951–0.991).

### 2.5. Data Analyses

The SPSS version 26.0 software (licensed by Khon Kaen University, Khon Kaen, Thailand) was used for data analyses. Statistical significance was set at *p* < 0.05. Demographic data of infants and parents were analyzed, and are reported as the mean, standard deviation (SD), frequency, percentage, minimum, and maximum. The AIMS scores were plotted on curves developed in our previous study [39] for full-term Thai infants, in order to obtain the gross motor development percentiles. The ranges of percentile from 14 assessments are reported as the mean (SD) and standard error of measurement (SEM). The ranges of percentile ranks obtained from the AIMS across the 14 assessments of each infant are visualized using box plots.

The General Linear Mixed Model (GLMM) was used to analyze the association between the gross motor percentile variability and associated factors, such as the demographics of parents, parental and socio-economic data, sleep position, and equipment usage during childcare. The GLMM can detect changes over time between individuals and within an individual. In the current study, ID and age were included as fixed effects, and related factors were treated as random effects. The analysis was coded for each time point (each month). For categorical data, infants with the reference factor or without the factor were coded as 0, and infants with the factor were coded as 1. A univariate analysis was performed first, in order to determine the relationships between associated factors and gross motor percentiles. Significant variables in the univariate analysis were used for the initial model. Backward elimination was performed, whereby factors were entered into the equation and then sequentially removed, in order to determine which factors explained the differences in the relationship between predictors and outcome variables. The factors that did not make a significant contribution to the model were removed and rechecked using the likelihood-ratio (LR) test [40].

## 3. Results

Forty-four infants were recruited; however, three infants dropped out of the study at 3, 7, and 9 months, as their families had moved. Data for the remaining 41 infants were then analyzed. Around 56% (*n* = 23) of these full-term infants were male. Table 1 shows demographic data for all infants. Infants were born by natural birth (*n* = 22; 53.7%), Cesarean section (*n* = 18; 43.9%), or forceps extraction (*n* = 1; 2.4%). The birth orders of infants were 39% firstborn, 48.8% second-born, 9.8% third-born, and 2.4% fourth-born. All infants were appropriately vaccinated during the 13-month period of data collection. The mean (SD) illness duration was 4.2 (2.8) days. Illnesses in infants consisted of fever, common cold, diarrhea, tonsillitis, acute enteritis from viral infection, and hand, foot, and mouth disease; however, no infants were seriously ill or admitted to the hospital.

**Figure 1:**
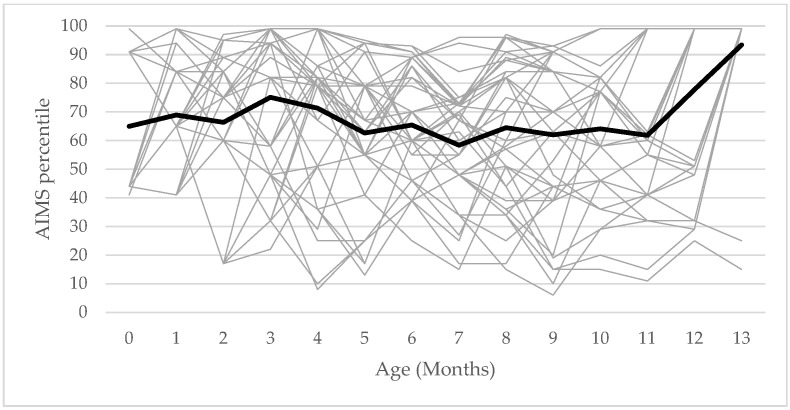
The up and down with a non-systematic pattern of gross motor percentile rank for the 41 participants. The gray lines show individual participants’ gross motor percentile. The darkest line shows the mean values of each month’s percentiles.

The longitudinal results showed that the gross motor development percentile did not follow a systematic pattern in infants from 0 to 13 months. Figure 1 shows the line of gross motor percentile rank in every month for the 41 participants. The percentile rank of each infant went up and down with a non-systematic pattern. The ranges of gross motor development percentiles were from 36 to 93 with a mean value of 65.95 (SD = 15.7; SEM = 2.3).

There were 4.9% of infants showing their percentile below the cutoff percentile of gross motor development at 4 months of age; however, they could walk independently at the age of 12 and 13 months, and their mean ranges of gross motor percentiles were 89 and 91, respectively. Thirty-eight infants (92.7%) could walk independently at different ages within the study period: 10 months (*n* = 2, 4.9%), 11 months (*n* = 9, 2%), 12 months (*n* = 15, 36.6%), and 13 months (*n* = 12, 29.3%). The remaining infants achieved independent walking after 13 months.

**Figure 2:**
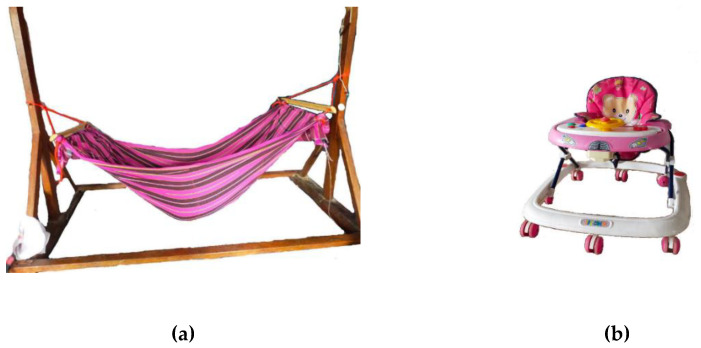
Childcare equipment: (**a**) Baby hammock; and (**b**) baby walker.

In each month of data collection, the main caregivers most frequently reported that the sleep position of the infant during the daytime was supine (60–100%), while side-lying was the most frequent position when infants were put to sleep at night (39–68.3%). The distribution of infants from 0 to 13 months who used equipment during childcare is shown in Table 2. Almost half of the families (43.9%) started to put their babies to sleep in baby hammocks after birth, and they continued to use them throughout the 13 months of observation (Figure 2). The mean (SD) duration of baby walker (Figure 2) usage was 4.1 (2.1) months with a range of 1–8 months. During the ages of 6–8 months, more than 80% of the 41 infants were placed in a baby walker.

Table 3 presents the regression results for the factors included in the model. The univariate analysis using the GLMM showed the relationship between the gross motor percentile and the analyzed factors; maternal and paternal education, household income, breastfeeding, equipment usage, sleep position during the day, and sleep position at night. Three factors remained in the final analysis: The use of a baby hammock, the use of a baby walker, and sleep position during the day (Table 4). The gross motor percentile was significantly positively associated with the use of a baby hammock (Coef. = 7.33; *p* = 0.04), whereas baby walker usage was negatively associated with the gross motor percentile (Coef. = −8.83, *p* < 0.0001).

## 4. Discussion

The aims of the current study were to: (1) Investigate intra-individual variability in the gross motor development of typical full-term Thai infants raised within their home environment and (2) examine the factors associated with the variability in gross motor development percentiles during the first year. The healthy full-term infants in this study displayed variable gross motor development percentiles without a systematic pattern. The mean (SD) percentile of gross motor development was 65.95 (15.7), which is in line with the result (66.8 (13.5)) of a previous study in 46 healthy Canadian infants [4]. This result supports the findings of previous longitudinal assessments indicating that typically developing infants exhibit variability in gross motor percentile from birth to 13 months [4,5,6]. Moreover, the gross motor percentile of infants in our study showed similar trends to those in a previous study [4], possibly due to the nature of the variability in motor development in healthy full-term infants. The widest range of the gross motor percentile was from 39% to 93%, while the widest percentile range in the study of Darrah et al. was from 34% to 87%.

Having a gross motor percentile lower than the cutoff point at a particular time does not indicate that the infant has a developmental delay. Darrah et al. [36] have reported that the cutoff point for the risk of delayed motor development in infants is below or equal to the 10th percentile at an age of 4 months and less than the 5th percentile at an age of 8 months. According to this cutoff point, we found that 4.9% of infants showed gross motor percentiles below or equal to the 10th percentile at the age of 4 months. Darrah et al. found that 31% of infants had gross motor percentiles lower than the cutoff points on at least one occasion [4]. They suggested that a low gross motor percentile could be attributable to a period in which few or no new motor skills were developed, compared with infants who mastered many new skills [4]. In contrast, the increases in gross motor percentiles in infants were expected, as a diversity of behavior acquisitions occur in the first two years of life [9]. Moreover, although two infants had gross motor scores below the 10th percentile at 4 months, they showed an overall wide range of intra-individual variation in gross motor percentile, and could walk independently within 13 months [1]. With cross-sectional observation, we cannot determine whether infants who showed percentiles below the cutoff are those with delayed gross motor development, as gross motor development displays variability depending on environmental factors and the movement experiences of the infants. This was in line with the longitudinal results of Pereira et al., who showed that variability in motor development is better explained by the environment [9]. Therefore, series assessments are required for screening gross motor development, especially during the first two years. In the case of infants who exhibit persistent below cut-off gross motor percentiles, standard assessment with reporting from mothers or caregivers, or play-based assessment across context, could be performed in order to obtain recommendations for appropriate childcare [7].

In this study, baby hammock usage demonstrated a significant positive impact on gross motor development. Regression analyses indicated that using a baby hammock could increase the gross motor percentile by 7.33 points. The baby hammocks in Thailand are often made of fabric that is soft but durable and can be easily tied to a stable frame. Most infants are put to sleep in a baby hammock during the daytime. Our result was contrary to previous research that investigated the effect of baby hammock usage on neuromotor development in 26 full-term infants at the age of 6 months [41]. This previous study demonstrated that full-term infants who used a baby hammock had significantly lower total AIMS scores, especially in the standing position, compared with those that did not use a hammock [41]. A conclusion cannot be drawn about the effect of baby hammock usage on gross motor development, as the previous study cross-sectionally assessed gross motor skills at 6 months, and the number of infants varied greatly between the two groups (9 and 17). Research on the effect of baby hammocks on gross motor development is still controversial. Moreover, there has been limited research directly examining the effect of baby hammock usage on gross motor development from a younger age; as such, further appropriately designed randomized controlled studies are needed to clarify the effect of baby hammock use on gross motor development.

Our longitudinal study revealed that using a baby walker is negatively associated with gross motor development. Regression analyses indicated that using a baby walker during the first year of life could lower the gross motor percentile by 8.83 points. The National Institute for Child and Family Development of Thailand also recommends avoiding the use of a wheeled baby walker during child rearing, especially in the first two years. Unfortunately, Thailand has no regulations on the sale, importation, or advertisement of baby walkers. Using a baby walker during child rearing is still common practice in Thai households. The infants in this study were put in a baby walker as early as 3 months of age. Infants may be able to move in different directions while they are in the baby walker, but the baby walker provides support around the thoracic level, which could impede trunk control and weight shifting in the upright position, especially during standing and walking [24]. Notably, more than 80% of the 41 infants were placed in the baby walker at the ages of 6–8 months. During this period, infants typically develop trunk control, which allows them to maintain upright positions, such as sitting, pulling to stand, or standing. Using a baby walker at the onset of motor milestones such as sitting, pulling to stand, standing, and walking could be one possible risk factor contributing to delayed trunk control required for upright positions.

Additionally, previous studies have reported the delayed onset of key gross motor milestones, including sitting, crawling, standing alone, and walking alone, in baby walker users compared with non-users [21,25,26]. In 2021, Bezgin et al. have reported the adverse effects of baby walker usage on trunk control and infant motor development. Infants using a baby walker showed significantly lower trunk control (assessed by Segmental Assessment of Trunk Control, SATCo) and less motor development (assessed by the AIMS) than those who did not use a baby walker [24]. On the other hand, Yaghini et al. have reported that baby walker usage did not show a statistically significant effect on gross motor development [42]. However, these researchers assessed gross motor development using the Ages and Stages Questionnaire (ASQ) at the ages of 12 and 18 months [42]. The researchers concluded that more studies should be conducted to confirm the results of the study [42]. The non-significant result of this previous study could be due to the age of the infants, which was 12 and 18 months; at this age, infants have achieved upright control in independent sitting and standing positions. However, usage of a baby walker in this age range could be appropriate with all-time supervision from parents or caregivers.

Our result strongly supports previous research evidence that baby walkers should not be recommended for child-rearing practices, especially during the first year [21,22,23,24,25,26]. The American Academy of Pediatrics recommends a ban on the manufacture and sale of mobile infant walkers. Confirmed by a great amount of research evidence, the baby walker has been banned in many countries due to the injury it may cause especially, in infants and toddlers aged less than 15 months [21,22,43,44]. Types of accidents could be the overturning of the baby walker (91.7%) or falling downstairs with the baby walker (8.3%). There have also been a lot of head injuries found in baby walker users [44]. A previous study reported that parents or family did not realize the danger of the baby walker use, and parents think that the baby walker may be beneficial when the family needs to perform household chores [24]. In addition, gait disorder, especially toe walking, was more frequently found in baby walker users [21,22].

Putting infants to sleep in the side-lying and prone positions during the day had a positive effect on the gross motor percentile, compared with the supine position; however, the results were not statistically significant. Sleeping in the prone position may promote the early achievement of gross motor milestones [20]. Devis et al. have found that infants who slept in the prone position reached several motor milestones earlier than those who slept in the supine position [16]. Similarly, Majnemer et al. also found that infants who slept in the prone position had stronger anti-gravity extensor movements at 4 and 6 months of age [15]. In contrast, in 2014, Darrah et al. argued that most parents placed their infants in the supine position, which does not result in developmental delays. There was no association between sleep position and gross motor development [17]. In Thai culture, infants tend to be put to sleep in a baby hammock during the day; therefore, we encourage parents or caregivers to put infants on their stomachs while awake during the day when infants are not able to roll independently. Moreover, parents should provide the opportunity for infants to move freely in various positions and explore environmental circumstances safely, which would promote their trunk control while experiencing movement during their first years.

Longitudinal assessments and analyses of the gross motor development percentile over 13 months allowed us to reveal the variability in gross motor development and factors associated with gross motor development; however, the current study has some limitations. The post hoc power of testing 41 infants was 0.59. The number of participants needed to achieve a power of 80% is 57 infants. The participants recruited in this study were only from the Northeastern region of Thailand, and the gross motor percentiles referred to the reference scores of Thai infants. Therefore, broad conclusions should be drawn cautiously. Furthermore, we did not record the time duration and frequency of equipment usage. We cannot draw a conclusion regarding the negative or positive factors affecting gross motor development. A further prospective analytical study is needed to compare the specific key motor development or postural control in the sitting or standing position between infants who are exposed to a baby walker and those who are not. Data on the duration and frequency of equipment usage and the associated adverse effects are required to estimate the effect of equipment usage on trunk control during the development of upright motor skills.

## 5. Conclusions

The development of gross motor skills in healthy full-term infants in this study fluctuated, and the percentiles showed no systematic pattern across longitudinal assessments. Our results support those of long-term assessments that clearly reflect variability in development, which is a result of the dynamic circumstances and experiences of movement during child growth. Placing infants in a baby walker during the first year of life, while infants develop their trunk control in the anti-gravity postural milestone, is not recommended. However, if unavoidable, it is suggested that infants be put in a no-wheel baby walker and supervised by a caregiver at all times. In contrast, putting infants to sleep in a baby hammock during the daytime may have a positive impact on gross motor development.

## Figures and Tables

**Table 1:** Demographic data of 41 infants.

	Mean (SD)	Min–Max
Birth length (cm)	52.0 (2.1)	47–55
Birth weight (g)	3214.1 (427.9)	2540–4210
Head circumference (cm)	33.5 (1.4)	31–37
Apgar score at 5 min	9.7 (0.4)	9–10
Gestational age (weeks)	38.8 (0.9)	37–40

**Table 2:** Number of infants (%) aged from 0 to 13 months who used equipment during childcare.

Number of Infants at Each Month	Baby Hammock	Baby Walker	Highchair	Playpen
0 (*n* = 41)	18 (43.9)	-	-	2 (4.9)
1 (*n* = 41)	34 (82.2)	-	-	1 (2.4)
2 (*n* = 41)	36 (87.8)	-	-	-
3 (*n* = 41)	38 (92.7)	2 (4.9)	-	-
4 (*n* = 41)	37 (90.2)	7 (17.1)	-	-
5 (*n* = 41)	37 (90.2)	20 (48.8)	-	1 (2.4)
6 (*n* = 41)	36 (87.8)	33 (80.5)	-	1 (2.4)
7 (*n* = 41)	35 (85.4)	34 (82.9)	-	2 (4.9)
8 (*n* = 41)	35 (85.4)	35 (85.4)	1 (2.4)	7 (17.1)
9 (*n* = 41)	33 (80.5)	28 (68.3)	1 (2.4)	12 (29.3)
10 (*n* = 41)	35 (85.4)	20 (48.8)	1 (2.4)	16 (39.0)
11 (*n* = 39) *	33 (84.6)	16 (41.0)	1 (2.6)	10 (25.6)
12 (*n* = 30) *	24 (80.0)	9 (30.0)	1 (3.3)	7 (23.3)
13 (*n* = 15) *	9 (30.0)	3 (20.0)	-	3 (10.0)

* Decreased number due to the fact that infants were not observed after they achieved independent walking.

**Table 3:** Factors affecting gross motor percentile (univariate analysis).

Factors		Coef.	Std. Err	z	*p*-Value	95% CI
Maternal education						
	≤High school	Ref				
	>High school	−0.38	4.57	−0.08	0.93	−9.35, 8.59
Paternal education						
	≤High school	Ref				
	>High school	3.60	5.16	0.70	0.48	−6.51, 13.72
Household income		6.40	0.0001	0.07	0.94	−0.0002, 0.0002
Breastfeeding						
	No	Ref				
	Yes	1.87	2.53	0.74	0.46	−3.09, 6.84
Baby hammock						
	No	Ref				
	Yes	7.90	3.65	2.17	0.03 *	0.76, 15.05
Baby walker						
	No	Ref				
	Yes	−8.34	2.02	−4.13	<0.0001 *	−12.30, −4.39
Highchair						
	No	Ref				
	Yes	−8.21	13.03	−0.63	0.53	−33.75, 17.33
Playpen						
	No	Ref				
	Yes	−0.38	3.41	−0.11	0.91	−7.06, 6.30
Daytime sleeping position						
	Supine	Ref				
	Side-lying	3.98	3.92	1.02	0.31	−3.70, 11.65
	Prone	3.41	6.38	0.54	0.59	−9.09, 15.92
Nighttime sleeping position						
	Supine	Ref				
	Side-lying	−1.12	2.30	−0.49	0.62	−5.64, 3.39
	Prone	3.07	3.77	0.81	0.42	−4.33, 10.47

* Significant value *p* < 0.05.

**Table 4:** Factors affecting gross motor percentile (multivariate analysis).

Factors		Coef.	Std. Err	z	*p*-Value	95% CI
Baby hammock						
	No	Ref				
	Yes	7.33	3.61	2.03	0.04 *	0.26, 14.40
Baby walker						
	No	Ref				
	Yes	−8.83	1.95	−4.52	<0.0001 *	−12.66, −5.00
Daytime sleeping position						
	Supine	Ref				
	Side-lying	2.99	3.80	0.79	0.43	−4.44, 10.44
	Prone	4.37	6.24	0.70	0.48	−7.86, 16.59

* Significant value *p* < 0.05.

## Data Availability

Not applicable.
